# “… and after That Came Me”. Subjective Constructions of Social Hierarchy in Physical Education Classes among Youth with Visual Impairments in Germany

**DOI:** 10.3390/ijerph182010946

**Published:** 2021-10-18

**Authors:** Martin Giese, Sebastian Ruin, Jana Baumgärtner, Justin A. Haegele

**Affiliations:** 1 Department of Natural and Human Sciences, Heidelberg University of Education, 69120 Heidelberg, Germany; martin.giese@ph-heidelberg.de; 2 Institute of Human Movement Science, Sport and Health, University of Graz, 8010 Graz, Austria; jana.baumgaertner@uni-graz.at; 3 Center of Movement, Health, & Disability, Old Dominion University, Norfolk, VA 23529, USA; jhaegele@odu.edu

**Keywords:** physical education, visual impairment, social hierarchy, feeling valued, bullying, inclusion

## Abstract

The aim of this study was to reconstruct subjective constructions of experiences in PE and feelings of being valued within PE classes in Germany by students with visual impairment (VI). Two female and two male students (average age: 19.25 years) participated in the study from the upper level. For the reconstruction of experiences of feeling valued, episodic interviews with a semi-structured interview guide were used. The data analysis was conducted with MAXQDA 2020 based on content-related structuring of qualitative text analysis with deductive–inductive category formation. To structure the analysis, the main category, feelings of being valued, was defined by two poles (positive feelings of being valued as opposed to bullying). As a main finding, respondents primarily reported negative feelings and experiences characterized by instances of bullying, discrimination, and physical and social isolation, perpetuated by both their peers and teachers. In search of a deeper understanding, we identified social hierarchy as an underlying structure determining the students’ perceived positioning within the social context and thus directing their feelings of being (de-)valued. It became evident that it is not the setting per se that determined social hierarchy, but that it is more about the concrete manifestation of social hierarchy.

## 1. Introduction

According to Article 24 (2a) of the Convention on the Rights of Persons with Disabilities (CRPD), “children with disabilities are not [to be] excluded from free and compulsory primary education, or from secondary education, on the basis of disability”. In international educational contexts, this statement is usually interpreted to mean that the realization of the right to full inclusion is tied to children with and without disabilities being taught together in the same physical class or environment [1]. The normative orientation of this educational policy [2] has been largely unquestioned in the international inclusion discourse for some time. As a result, homogeneous educational placements have largely been disbanded all over the world in order to include all students in general education, including in physical education (PE) [3,4].

Specific to children with visual impairment (CWVI), McLinden et al. [5] (p. 180) state that within Anglo-American contexts, “the majority of children and young people with vision impairments but no additional disabilities are now educated in mainstream settings.” In the USA, for example, CWVI are now largely enrolled in general education and neighborhood schools [6], whereby schools for CWVI have shifted from serving those with visual impairments and no other disabilities to those with multiple disabilities. However, this does not apply to Germany where the interpretation of Article 24 (2a) of the CRPD is highly controversial and there is a vehement dispute about how to interpret the CRPD [7]. At present, almost all federal states in Germany continue to prefer to maintain two educational systems, particularly in the case of sensory disabilities [8]. The argument for segregated schooling is grounded in the belief that this setting provides a more beneficial and appropriate environment for children with disabilities. However, regardless of the educational placement or setting in which CWVI are enrolled for PE, they should be supported in ways which allow for them to *feel included* in their classes [4].

Here, we use the phrase feel included in alignment with conceptualizations of inclusion suggested by Stainback and Stainback [9] and adopted by Spencer-Cavaliere and Watkinson [10], where inclusion is thought of as a subjective experience that is centered on feelings of acceptance, value, and belonging. In this paper, we focus specifically on feeling valued. According to White and Mackenzie-Davey [11], feeling valued can be seen as a positive emotion centered on how others evaluate an individual in relation to some ability or characteristic that they consider to be important. Within this conceptualization of ‘feeling valued’, there is an important distinction between knowing that one’s work or abilities are valued, and feeling as though they are, and the latter is largely related to some form of praise or recognition from others within a social context [12]. In studies examining experiences within PE, and other educational contexts, the characteristic of feeling valued supports the importance of the role that others, such as peers or teachers, play in individuals’ feelings of being valued.

Unfortunately, pedagogical research in PE focused on supporting the inclusive agenda is often conducted about people with disabilities and tends to emphasize the voices of stakeholders and professionals, while overlooking the voices of those with disabilities [13,14] (p. 68). Research activities on PE refer to the perspectives of parents [15], peers without disabilities [16], or teachers without disabilities [17]. In this respect, it appears that research is largely focused on understanding stakeholders’ perspectives about inclusion, rather than CWVI feeling included [18]. This is problematic, as it limits our understanding of the thoughts, feelings, and experiences of CWVI themselves, which are central to interpretations of inclusiveness [19], and conflates concepts associated with ‘inclusive placements’ and ‘inclusive experiences’ [4], of which the latter must be uncovered by speaking with CWVI themselves about their experiences [20]. By centering research on the voices of CWVI, researchers can help amplify the voices of CWVI while gaining an understanding of the particularities of their experiences, and how those particularities influence how CWVI construct their social experiences in PE. This viewpoint is well-aligned with calls for academics to amplify the voices of persons with disabilities in research about inclusion [21,22], as well as the ‘nothing about us, without us’ movement which emphasizes the empowerment of persons with disabilities through centering their experiences [23,24].

However, the few studies that have explicitly considered the perspective of CWVI suggest that overwhelming negative experiences may very well occur in integrated settings [25,26]. In line with these findings, Ball et al. [27] pointed out in their systematic review that bullying of CWVI by peers and teachers is a widespread phenomenon in PE, which leads to social isolation. These findings are also consistent with assertions by Atkins [28], who suggests that educational frameworks intended to promote inclusion can, at an individual level, provoke negative experiences where people with disabilities subjectively evaluate their individual experiences differently than educational policy frameworks intend. As such, despite the positive intentions that inclusive settings may be derived from, subjective experiences associated with inclusion (i.e., belonging, acceptance, and value) appear unavailable to many CWVI in these settings [4]. In this sense, further research explicitly exploring the perspective of CWVI is critical and can further contribute to our understanding what individual needs result from it [22].

Against this background, the focus of the study lays on understanding CWVIs’ subjective perceived valuation and in locating them in the sensed social context in PE. Thereby, inclusiveness is not understood based on whether someone is included or excluded in certain activities, but linked to the subjective assessment of whether the particular students feel valued during what is happening in PE—this is congruent with the claims of disability studies. In view of the above-mentioned points, this research project sought to understand CWVIs’ subjective experiences about the emotional spectrum of feelings of being valued to being disvalued (e.g., bullied) in PE classes in Germany, in order to promote awareness (Article 8 CRPD) as to how joint school settings can be designed to be as inclusive as possible for students with and without VI [24]. The outlined considerations gave rise to two main research questions:

- How do CWVI understand the social context within PE?
- How do CWVI understand feelings of being valued within PE?

## 2. Materials and Methods

In order to understand the subjective experiences of CWVI in PE, this explorative study used qualitative survey and analysis methods. By assuming a research perspective which seeks to access subjective viewpoints [29], the intention was to reconstruct specific manifestations of the participants’ social reality (the perspective of CWVI) in certain situations (experiences in PE classes) [30]. This means that this study aimed at understanding CWVI’s specific and individual viewpoints and constructions within the PE setting they were situated in. This provides a deeper understanding of the connection between factually existing situations, subjective feelings and the respective way of dealing with them. A quantitative approach could not reveal these connections, and therefore a qualitative approach was adopted so that the participants’ narratives could emerge. According to Denzin and Lincoln [29], qualitative research is a situated activity that locates the observer in the world and allows us to study things in their natural settings, attempting to make sense of or interpret phenomena in terms of the meanings people bring to them.

### 2.1. Sample

The data were collected in January 2021 at a privately owned state-approved special school for CWVI in Germany. This school pursues a school concept with the same objective of the general higher education entrance qualification (known in Germany as Hochschulreife). This is the only upper secondary school for CWVI aimed at receiving an undergraduate degree in the German-language area. Due to the large draw area, the school is attended by nearly all students, with very few exceptions, as a residential school. On the whole, two female and two male students between 19 and 20 years (average age: 19.25 years) participated in the study from the upper level (Table 1). All participants gave informed consent, and their names were changed to pseudonyms for the narrative reconstructions and verbatim quotes. All participants plan to take the centralized school leaving examination (Abitur) and were attending the residential school at the time of the data collection.

All respondents were officially classified as visually impaired or blind. The criteria for visual impairment according to German social law include three degrees of visual impairment: VI is defined as a visual acuity between 0.3 and 0.05 in the better eye with the best possible correction. Severe VI is defined as a visual acuity between 0.05 and 0.002 in the better eye with best possible correction. Blindness is defined as best-corrected visual acuity of 0.02 or less in the better eye or when the visual field is less than 5 degrees. One participant, Tim, also had a hearing impairment. Tim was the only participant who continuously attended a special school during the entire secondary education first stage (ISCED) 2, (for international understanding, we use UNESCO’s terms of the International Standard Classification of Education), whereas the other three participants were educated at mainstream schools close to their homes during ISCED 2 and explicitly decided to switch to the special school at the transition to the secondary education second stage (ISCED 3). The composition of the sample reflects the school reality of CWVI in Germany, as discussed earlier in this article.

It is important to note that all students attended the special school for CWVI at the time of data collection. However, except for Tim, all participants were enrolled at mainstream schools close to their homes during the entire ISCED 2. Tim was schooled at a different special school for CWVI during ISCED 2. However, all the participants explicitly decided to switch to a (new) special school at the transition to ISCED 3 and with it consented to the associated boarding school residence. For this particular study, the participants report exclusively about their time during ISCED 2.

### 2.2. Data Collection and Analysis

For the reconstruction of experiences of feeling valued within PE classes, episodic interviews with a semi-structured interview guide were used [31]. This strives for a ‘changes from the respondents’ viewpoint but without exclusively focusing on the biographical processes’ [31] (p. 278). The episodic interview aims for ‘stories about situations in which interviewees have had certain experiences’ [31] (p. 274); it was also chosen because the interviewer works as a teacher at the school in addition to his function as a researcher and had already known all of the participants at the time of the survey. At the time of data collection, there was no teaching relationship between the students and the researcher. To avoid socially desirable responses and ensure confidentiality in each interview, participants were told that their statements would be strictly anonymized and that they were not expected to emphasize certain perspectives. The personal and confidential relationship was intended to allow a deeper access to the individually relevant barriers in the mainstream school system, which manifested in the episodic stories. In addition, being a teacher specialized for CWVI provided knowledge about CWVIs’ situation, which allowed an appropriate conversation management within the interview.

Every interview was digitally recorded and transcribed. The data analysis was conducted with the MAXQDA 2020 software based on content-related structuring of qualitative text analysis with deductive–inductive category formation; this is explicitly recommended for the evaluation of episodic interviews [32]. In a systematic explication of the researchers’ and respondents’ prior knowledge with the procedure proposed by Ruin [33], the main category “feelings of being valued” arose. This category was defined by two poles (positive feelings of being valued opposed to bullying), which opened up a spectrum. This figure guided the analysis. Connecting this theoretical lens with the outlined social contexts, we identified social hierarchy as another main category. Social hierarchy emerged as an underlying structure determining the students’ perceived positioning within the social context, and hence directing feelings of being valued. Within this iterative process, subcategories were identified inductively as aspects of social hierarchy and differentiated using the transcripts in a cyclic process [34]. These subcategories are (1) social hierarchy in between students, (2) social hierarchy between students and teacher(s) having another quality (e.g., due to authoritative figuration), and (3) social hierarchy regarding power not directly lying in the hands of the social actors in PE lessons. This step and the in-depth analysis were carried out for each case and on each interview separately. As a result, the two main categories, ‘social hierarchy’ and ‘feelings of being valued’, and the link between those two were identified to be the focus of the results section of this study.

## 3. Results

In order to depict the individualized experiences of each of the participants, as well as salient features of these experiences that influenced feelings of being valued, the results section is presented at the case level. That is, narrative depictions, or reconstructions of each participant’s experiences, are provided separately in order to provide a deeper understanding of the connections between the categories and the different connotations on the individual level. This allows us to highlight the particularities of each individual participant’s experiences, while also working within alignment of the purpose of the study, as well as the main categories that were identified throughout the data analysis process.

### 3.1. Carla

When reflecting on her experiences in PE during her time in integrated schools, Carla largely recalled experiencing ongoing discrimination, bullying, and harassment in PE both by teachers and students during lessons. In her interview, she described that instead of being supported, she was staged as not having the mental capacity to attempt tasks, and she pointed out a lack of understanding of her impairment and valuation of her as a person by teachers. Reflecting the views of the teachers, students treated her as less worthy than the rest of students and devalued her to the point of purposefully physically endangering and hurting her by manipulating safety measures on sport arrangements. Peers even filmed an event where they manipulated gymnastics equipment which led to Carla breaking her wrist. Carla recalled this instance with great sadness, noting that her peers appeared to take pleasure in watching her become injured by their harassment. This event escalated to where police were brought into the school to investigate why students manipulated the equipment for Carla to become injured, and to settle a dispute about a video that was recorded and shared throughout the school of the incident.

The climax of her PE experience was that this treatment toward her led to the official solution of discarding her from PE, rather than adapting the lessons or changing behavior towards her. That is, her integrated PE experiences were ended by her ban from PE explicitly showing her as unvalued in PE and causing the inability to enter the social hierarchy system at all. Carla states that generally she actually enjoys participating in sports but could not find herself enjoying PE at all. She considers the ban a shame, but simultaneously she was relieved to be out of the lessons where she was constantly being disregarded and devalued.

*“Good at sports, not good at sports. Popular, not popular. And popular and good at sports were of course at the top. After that came popular and not so good in sports. And after that came not popular and good in sports. And after that came not popular and not good at sports. And after that came me”.*

(Carla, pos. 45)

When discussing her social status within her PE classes, Carla provided the preceding narrative, which explicitly describes where she found herself socially within her class. Essentially, Carla described herself as “not as part of the class social hierarchy” but rather outside of and perhaps below it. According to Carla, she was not even considered to be a peer within her class and was thought of to be in a different category, by herself. Carla’s status below the social hierarchy, subhuman in some respects, materialized in challenging interactions with stakeholders within her PE classes due to a lack of value towards Carla. According to Carla, negative experiences occurred consistently, “actually every sport lesson” (ibid., pos. 13), and were typified by instances of marginalization and othering, such as being selected last when picking teams. When reflecting on these experiences, Carla suggested that to some degree these instances should be expected, since she was not within the same social status as her peers and could not perform well because of her visual impairment. She further noted that PE in particular was a challenge, where “I think that is just in PE, so, that is in no other subject like in PE is so striking, how different the students are” (ibid., pos. 79). This statement supports the notion that PE constructed its own social hierarchy, that may have been independent of other subjects, and was at least to some degree associated with her capabilities to perform and valuation of her actions.

Situations escalated at times into instances of bullying and harassment, where she was viewed as nothing more as an object of entertainment for her peers and she felt as though she was identified and devalued by her peers because of her skills or disability. Instances where other students purposefully endangered Carla to physical injuries by exploiting her limited visual abilities obviously embody the peers’ concept of her being entertainment, rather than a human being that may be hurt or injured by them. As such, her peers displayed a clear lack of empathy and sense of responsibility. For example, Carla reflected on a second instance where equipment was manipulated to injure her, where the “lever for securing the bars was mysteriously not engaged at the bottom”, demonstrating how her peers viewed her as no more than entertainment for them, and devalued her as a human being.

Carla’s experiences with her peers are likely a reflection of her experiences with her teachers [35]. Carla reported that she felt unhuman to her teachers, who did not appear to value her as a student at all. This feeling was informed by a number of instances, such as when her teacher taught her in a demeaning manner in front of her peers and made a joke of her inability to see specific lines on the ground. This public instance staged not only her disability but also disputed her mental state, and did not encourage her peers to help her in PE but rather supported the idea of using her as an entity of entertainment.

*“[…] and then she basically said in front of the whole class, well, here’s the line, take a look at it […] she said to me, look, you can’t see it, sit down and feel it. So, it wasn’t this, come on I’ll help you, I’ll show you where the line is, but […] sit down and make a fool of yourself. […] As if I’m just too stupid for it”.*

(ibid., pos. 29)

This quote also demonstrates that while fellow students assisted each other during exercises, Carla was left alone and only sometimes was assisted by the teacher, thus promoting the distancing between the students even further.

At the end of the day, Carla ended up being discarded, literally, from the PE environment because her teachers no longer wanted to deal with the risk of her presence. More specifically, Carla recalled that

*“there were really teachers who didn’t want to teach our class because I was in there, because they were just afraid of it because of visual impairment”.*

(ibid., pos. 57)

Eventually, her status as being excluded from the social hierarchy of PE were formalized, where she was told “please do not even go to the gym” and that she “was not allowed in there anymore” (ibid., pos. 17). She reflected on this, noting that

*“on the one hand, I was of course very happy, because then I didn’t have such things anymore. And on the other hand, I thought it was a shame, because I actually like sports, too”.*

(ibid., pos. 19)

### 3.2. Jonas

Until 11th grade, Jonas continuously attended mainstream schools. Being asked about his experiences, Jonas describes most of his school experiences in PE as very negative. Particularly in large sports games, but also in lessons that required a quick reaction speed, he was regularly banished by the teacher to the bench or to the storage room, where he was supposed to perform exercises with the TheraBand by himself (Jonas, pos. 15). By excluding him from the main student body, it seems as if he—similar to Carla—was never even considered a valued participant and a part of the social hierarchy system in PE. His situation did slightly improve after it was suggested that he could train on his own in a nearby weight room during regular PE classes. This was offered to him by a teacher, who said “if you want, you do not always have to sit on the bench, but you can also simply train in the weight room while we have PE” (ibid., pos. 13).

Astonishingly, it was completely clear to Jonas that neither the teachers nor his classmates could have done anything about his negative experiences, nor did they bear any responsibility for this situation of being out of the social hierarchy. In his perception, the problems lie in the size of the group and in the curricula (In Germany, the federal states are responsible for educational policies. Every federal state publishes statewide curricula, which are to be understood as *compulsory* guidelines that clarify what content must be addressed, in which grade and—at least to a certain extent—in what form. Teachers are obligated to follow the curricula) requirements, and therefore the responsibility does not lie directly in the hands of the social actors in PE: “Well, I mean, they have certain guidelines that they have to follow” (ibid., pos. 57). He considers the peers’ intentions to conduct “normal PE“ (ibid., pos. 59) to be absolutely legitimate and emphasized that the overall situation could not be changed “all at once because of one student” (ibid., pos. 119). Against this backdrop, his feelings of being valued in PE seemed to be ambivalent. On the one hand, Jonas described that he had not experienced any bullying and “that most of my classmates were helpful“ (ibid., pos. 29). On the other hand, he also reports that classmates, with the exception of individuals who were very close to him, did not show any consideration for his specific situation in PE class, but he did not call for this as he states:

*“Sure, I can say now that they shouldn’t have thundered on it like that during volleyball, but I can’t ask that of my classmates if they want to do sports normally. […] It’s logical that they don’t all take me into consideration”.*

(ibid., pos. 83)

Jonas also described a similar perception regarding lesson content. He described that there were lesson contents with which he could cope better (e.g., individual sports), but he also clearly stated that no consideration or changes could or should be given to his needs (ibid., pos. 59). In addition to the active exclusion of Jonas, which has already been described above, it should also be pointed out that Jonas, without it being discussed with him, was excluded from grading in PE classes (ibid., pos. 35). Additionally, even though he could not make any sense in it, since he had to come anyway, he evaluated the waiver of the grade positively (ibid., pos. 41). Overall, the paradoxical finding emerged that Jonas described massive exclusions (extra rooms, no grades, no accommodations, etc.) and lamented his dissatisfaction with his PE classes; however, at the same time, he described that it would not be appropriate to make changes to the system just because of him. To him, the responsibility for the unsatisfactory situation was out of his, his peers’, and his teachers’ reach, so that there was nothing that could be done to change this preset organizational framing, which is why he also seemed to have no right to demand for his needs: “Maybe they should not have sent me off to a storage room. As stupid as that sounds now. However, I cannot blame them for anything else. Because I am one of 20 or 25 […]” (ibid., pos. 89). Later in the interview he commented: “It is clear that if you have a disability, whether it is with your eyes or if you have a crooked foot, that you’re at a disadvantage. However, there is nothing you can do about it. So, you are just the one who cannot” (ibid., pos. 119). Throughout the interview, Jonas did not state a clear position for how he accepts this framing, or he wants it to be changed.

Since Jonas believed his perspective was not valuable and that, in our eyes, legitimate claims for an adjustment of the PE class could follow from this, the only way left for him seemed to be the deserved and self-inflicted exile. It became clear to what extent Jonas has internalized his assigned position and his being at mercy to the others:

*“I usually withdrew when I noticed that they weren’t willing to look at me in quotation marks. Or here and there to go into a little more detail about me. Then, I just withdrew. So, on the bench or in the storage room”.*

(ibid., pos. 85)

In the end, it looked as if Jonas was trying to save his dignity by emphasizing the impossibility for any change. The responsibility for the situation was directed to a power not lying in the hands of the social actors in PE lessons. Through the unspoken agreement between the social actors banishing responsibility for the unsatisfactory situation from PE class, the social situation was superficially pacified. Simultaneously, the production of disability and the construction of an individual of another worth in social hierarchy and thus the exclusion from social hierarchy were thereby reproduced. This interpretation is somehow consistent with Jonas’ impression of his own non-existing position in the social hierarchy in PE. However, according to him, the social hierarchy in PE is determined by different parameters. For example, he reported that at the private school he attended, the socio-economic background of his classmates also played an important role. In addition, general athleticism, but also specific motor skills were crucial when club athletes were able to achieve above-average performance in the major sports games, for example. However, the degree of visual impairment also played an important role here, with lower vision correlating with a lower social status in the social hierarchy.

*“Well, if you were allowed to choose yourself, for example, if you had to make settings, then it was clear that the cool kids would all come together. Or the ones who play in the club and are super cool. Which is actually normal in PE”.*

(ibid., pos. 97)

### 3.3. Martha

Martha described herself as being very interested in sport and movement, and she reflected that PE used to be her favorite subject in school. She felt accepted by her peers not least due to her physical performance and she loved to compete with peers—even with the “male soccer players”, who in her eyes were the ideal of sporty guys. In 11th grade she became visually impaired, which was a surprise to her as well as to her family, peers, and teachers. At the time of the interview, she had been visually impaired for two years, and she did not accept her impairment. She suffers from making constant comparisons between how things used to be for her prior to her visual impairment, and how they are now. Furthermore, she has the feeling that her peers and teachers can hardly understand what consequences come along with becoming visually impaired by surprise, and what this change means to her. In the interview she reflected about her experiences of being a visually impaired girl in a general school, which she attended until 11th grade. The way she told her story gives a hint that the idea of the “normal life” she led at the same school before this dramatic change in her life informed her perceived descent in the social hierarchy of her school after becoming visually impaired. Within this complex structure, different situations along the spectrum of feelings of being valued and bullying arose.

In this interview it is striking that Martha did not feel valued by adults who were responsible for her school education. She described several situations where professionals delegated the responsibility for the situation to external factors, rather than trying to change something in their own behavior, in pedagogical work or in the organization of lessons and school. Due to a lack of support, teachers in her opinion did not value her in PE. She made clear statements on this during the interview, such as: “Additionally, then I was not allowed to participate in any sports. So, I just sat on the sidelines, because it was too dangerous for the teachers, because they did not want to take over the responsibility and liability” (Martha, pos. 3). In the end, she had the impression that for the teachers “[…] it just was not worth the effort […] to really think of something where I could have participated” (ibid., pos. 33). With one teacher, she experienced a highly problematic key situation in which she felt that she was even made responsible for being visually impaired and that she was somehow disturbing the PE lessons:

*“I remember sitting on the bench once, because I wasn’t allowed to participate, and the ball was kicked by someone who couldn’t play soccer very well, and it flew into my face. And I had the entire side red, and it was also really blue afterwards. And yes, the teacher said it was my fault, because I could have looked, and I should have heard where the ball was. And that’s why it was my fault”.*

(ibid., pos. 19)

Perfidiously, the same teacher obligated Martha to come to each lesson, and to sit there on the bench not taking part of the lesson for half a year (ibid., e.g., pos. 3, 29, 33). The way she repeatedly turned to this point in the interview gave the impression that this constant feeling of being devalued was a formative experience for her. Finally, she had never been given permission to participate in PE since she had an impairment. By being obligated to come and sit beside every lesson, it seems as if her disability and thereby her perceived low position in social hierarchy and her devaluation were staged, and therefore presented and highlighted to everyone again and again.

Looking back on the time when she had full sight, Martha reported that she felt accepted by her peers because of being sporty—not least due to her skills and the ability to compete with other sporty students (ibid., pos. 43). However, then, becoming visually impaired, she started to feel the impossibility of being part of the (sports) game or of a successful team due to her impairment: “it’s just a game, but you still do not want to lose” (ibid., pos. 55). This means that at least within the game, her peers would not accept her as an equal partner. However, still, when in certain activities she could add to the team’s success—and therefore felt like contributing value to the game—, she felt accepted and respected because her team was able to win with her (ibid., pos. 47). Here, it was evident that in her opinion, the logic of linking sport-related skills to the positioning in social hierarchy is something common and important for her peers—and also for herself. This link between social hierarchy and ability seemed even more underlined as she stated that within this context, “[a]fter all, I have not accepted my visual impairment until today” (ibid., pos. 73). It seemed that somehow, she still sticks with her “old” reference system in which the abled are the more valued, even if this means to value herself less.

Interestingly, Martha recalled being highly appreciative when she received accommodations so that she could participate in PE class. These accommodations allowed her to play an important role and feel valued in PE again (ibid., pos. 39) as long as her disability was not staged because of the accommodations (ibid., pos. 73, 75). For her, things improved when accommodations allowed her to take an active part in the PE class again and peers came up to help her, letting her feel that she was accepted the way she was (ibid., pos. 73). A key moment for this change was when in this PE class, peers criticized the accommodation given to her, but the teacher took over responsibility and explained the importance of the accommodation:

*“And I had a special shuttlecock for myself—my PE teacher had bought it with her own money—so that I could see it better, because I didn’t see this white one at all. And then a lot of people said, ‘Oh, that’s totally unfair that she has a colored one. Then, she can see it much better’. And then my sports teacher explained, this was true but I’d see really worse anyway. And only like that, I can see the ball at all. And then we also showed that with a normal, white ball that I just can’t see it. And I think I did hit it once. And afterwards I played really good badminton. And I think that’s just the point that if you explain to the students why I have this, show them why this disadvantage compensation is there, that this works a lot better”.*

(ibid., pos. 67)

These explanations by the teacher seemed to initiate a process of understanding by the peers and led to the change in their behavior described above—somehow, the teacher initialized a change in social hierarchy within the peers. Therefore, she emphasized the importance of respect and understanding by the teacher, as well as by the peers (ibid., pos. 73). She finally understood this when her new teacher treated her in a “new” way and helped her peers to understand her situation. In the end, it looks as if here, she felt valued as she is and (re-)started to value herself in a brighter way. Consequently, this opened up possibilities for contributing value to PE.

### 3.4. Tim

During Tim’s interview, he mainly focused on reflecting about his previous experiences at a different special school for students with VI and other impairments, where he had less usable vision than many of his classmates. In this setting, the range of visual ability was quite diverse; some of his classmates could almost see perfectly, whereas others were completely blind. Despite being at a special school, he experienced ongoing situations in PE where the lesson content was not adapted and left him little room to participate successfully. He voiced his concerns and anger over the fact that these inappropriate practices that restricted his participation and often devalued him. Confronted with an exercise (such as target throwing) which is constructed in a way that makes it hardly possible for him with his impairment to participate in experiences perceived to be meaningful, he spoke up for himself to his teachers, though mostly to no impact. Seeing no other way out of impossible exercises, Tim initiated a medical certificate excluding him from being graded in some sport practices. Ultimately, he would often stand passively on the sideline, while his peers were physically active. Occasionally, teachers would find and introduce successful adaptions in ball games, but generally no major changes were integrated. Often situations were “unfavorably solved” (Tim, pos. 9), leading Tim to the impression that adjustments might be too complex or unwanted, and thereby suggesting to him that he was not considered to be worth making the effort. In contrast, he voiced his need for recognition and consideration of him in his situation, marking the extent of how problematic the situation was for him in PE. Further, he explained that he mostly did not see solutions for participating in more demanding sport practices himself, indicating that the lack of knowledge might lead him to accept his fate in some situations. Adding to his frustration of being overheard, he describes that it is not his responsibility to always speak up for himself:

*“I mean, whether that would have been my job as a student to pick on it every time. Because it’s also exhausting, and somewhere I said to myself: ‘Actually, they should take care of it. That’s their job. How can you integrate people?’”.*

(ibid., pos. 55)

Within further statements, it became evident that speaking up for his needs was not just exhausting on a personal level, but also positioned him unfavorably within the social hierarchy system on the opposite side of the successful students, leading to an unwanted social conflict. Unadopted subject content seemed to force him, as an impaired student, to choose between speaking up for himself or playing along and participating in the construction and reproduction of inability. However, speaking up for himself would simultaneously mean risking to annoy the sportively successful and socially acknowledged students. Tim quoted other students saying: “Why are you complaining? You always have to […] again and again. Additionally, everything was great” (ibid., pos. 57). Regarding this mélange, Tim strongly described his feelings over perceived unfair treatment and lack of valuation, and generally sought for reassurance of his peers. Since the successful students had no interest in any adaptions and the other students would also position themselves unfavorably by speaking up, it is unsurprising that “from the others came no support” (ibid., pos. 57). He indicated that skills are of higher value in PE than just giving an effort when he recalled the prizes of a school sports festival—one prize based on skill level and an additional prize for effort. Therefore, underlining this social hierarchy:

*“And there was a medal of honor for total effort. I even got that once. It was pretty cool, but second place would have been even more exciting”.*

(ibid., pos. 21)

Furthermore, he stated that a sub segregation formed between students, where performance and grade were linked to the degree of visual ability which, to him, was mostly caused by the external factor of a predefined unfitting lesson content and could not solemnly be traced back to sportiness. Mostly he believed—similar to Jonas—that the department of education was responsible for unfitting content (since the teachers do—overtaking their perspective), but he also described that the teacher could have a slight impact (ibid., e.g., pos. 13, 19, 51) and that teachers could also influence the formation of the social hierarchy among students. In his opinion, the curricula should give everyone the opportunity to be successful and thus provide the chance to be a valued participant. To him, as the curricula specifications were, they did not seem to give room for variations in lesson topics, and thereby put him in social exile, constructing and staging his inability. Because of this deprival of entering the social hierarchy system and feeling valued in PE, he requested that:

*“maybe it’s not even the intention of the teachers. Because, I mean, the teachers mostly just follow instructions, I’ll put it that way. Something would really have to come from the Ministry of Education that this would be allowed. So, because the argument of my teachers, whom I had asked about it, was of course: ‘It’s in the curriculum.’ And of course you have to implement a curriculum. However, I think it must also be possible on the part of the Ministry of Education that visually impaired people or people with disabilities in general can deliver a performance of equal value”.*

(ibid., pos. 51)

Within this statement of Tim, his perceived establishment of social hierarchy and reciprocally corresponding spectrum of feelings of being valued could be comprehended as a cascade. Ultimately, as the curricula specifications are (referring to social hierarchy regarding power not directly lying in the hands of the social actors in PE lessons) content constructions in PE, often in line with teachers’ instructions (referring to social hierarchy between students and the teacher), provoke the establishing positioning of students within the social hierarchy system of peers (referring to social hierarchy in between students). The acceptance for his own situation in PE is grounded in his own doubts (and those of other students) of possibly integrating himself successfully in a certain practice and the inability of him and his teacher to change the PE contents. Tim justifies the behavior of all local participants by being reliant on the determinations of the (inaccessible) curricula, which to him, have to be changed in order for all impaired people to deliver a performance of equal value.

In this respect, Tim stated that a sense of cohesion is a central point in PE, especially regarding positive feelings of being valued. He emphasized the promotion of cooperation instead of advocating competition. To him acting against each other symbolized the general practice in PE, creating social tension and therefore his problematic standing. Interestingly enough, he described a setting in his current special school where he had an advantage due to his level of visual ability and therefore fulfilling his desire to be part of the game and to actively take a position within the social hierarchy of the players. In this instance, he could have decided to eliminate his advantage by wearing a blindfold like the others but decided against it.

*“And there I saw sometimes, […] a huge advantage. And that’s when I first noticed, I have to say, a form of freedom. That you were now really someone who could really play along. And there I have perhaps also sometimes strongly, I thought, times exploited. However, evenly also so more after its borders as one could […] And that’s when I first noticed what it was like to see well in quotation marks”.*

(ibid., pos. 63)

## 4. Discussion

This study sought to understand CWVIs’ subjective individual constructions of experiences in PE and feelings of being valued within PE classes in Germany. To structure the analysis, the main category feelings of being valued was defined by two poles (positive feelings of being valued opposed to bullying), which opened up a spectrum. Within this bipolar structure, many of the participants’ subjective experiences in PE were typified by challenging instances of bullying, but also of discrimination and physical and social isolation, perpetuated by both their peers and teachers. In search of a deeper understanding of these relations, we identified social hierarchy as an underlying structure determining the students’ perceived positioning within the social context and thus directing their feelings of being (de-)valued. Generally, the experiences reported in the interviews are closely aligned with those recalled by persons with disabilities in integrated PE settings in international contexts [35], including those with visual impairments [4,18], as well as those in previous German studies [25,26]. Interestingly, these negative experiences were not restricted to only integrated PE settings, whereas Tim, who exclusively attended special schools throughout his educational experiences, also experienced social isolation. As such, it is clear that hierarchies built within integrated settings, which are largely constructed based upon perceptions of ability [36], can also manifest in self-contained settings such as special schools for CWVI, where impairment level may play a critical role in where students find themselves in this hierarchy [37]. With that, it appears that there were few instances where our participants recalled feeling included within their PE classes, regardless of the educational setting in which they participated.

Inclusion is more than an individual’s quantifiable existence within an educational context, and conceptualizations of inclusion must extend beyond materiality and consider personal, subjective experiences within that space [38]. As such, we have adopted the conceptualization of inclusion as a subjective experience, and the concept of feeling valued is one of the central tenets of this conceptualization. Disappointingly, few instances of feeling valued were explicitly recalled among the participants in this study when reflecting about their PE experiences. The lack of feeling valued manifested in a number of ways in this study, including devaluation by peers as well as participants reflecting that their PE teachers demonstrated a lack of value because they were unwilling to reconsider pedagogical practices in order for them to participate, and rather than inconveniencing the class activities, participants were asked (or told) to sit out of activities and watch from the sideline. Clearly, here, teachers did not position themselves well to support feelings of being valued among participants by showing that their capabilities were valued, but rather reconstructed and supported a deficit understanding of impairment where they emphasized what participants could not do and removed them from activities [39]. It is important to recognize, however, that feeling valued is a fluid concept, that can change based on moment-by-moment expressions of value of one’s ability [12]. This was apparent in Martha’s experience, where she expressed feeling devalued in her PE classes after becoming visually impaired; there were instances where she received accommodations where she was permitted to help her classmates and team succeed, contributing to their success and her value. As such, she experienced a limited sense of value throughout her experiences. Not surprisingly, sometimes feelings of being valued seemed to occur in an ambivalent way for the respondents, as the interview with Jonas exemplifies. On the one hand, he describes his classmates more or less as “helpful“ (pos. 29) in PE, whilst on the other hand, he simultaneously identified a lack of consideration for his specific situation by them.

Further, a student’s feelings of being valued are inextricably linked to the place in which they believe they fit in the social hierarchy within their classes. We identified social hierarchy as an underlying structure in the interviews; it appears elementary and might be changed, but can never be overcome. When discussing the social hierarchy that existed within their PE classes, the participants recalled that this hierarchy was directly and clearly linked to their abilities to meaningfully contribute to the competitive nature of their classes. That is, because each of our participants described being unable to participate in many activities, being asked to leave PE activities, or not being offered accommodations in activities, they felt as though they were demoted in their class social system. This finding is consistent with those from Fitzgerald [36], who reported that participants in her study who participated in adapted sport (i.e., Bocce) did not receive the same social capital as their non-disabled peers because of the value that adapted sport played in the classes’ social ecology. As such, it is not surprising that our participants recalled feeling as though they were at the bottom, below, or completely not a part of the social hierarchy between the students. Carla puts this first aspect of social hierarchy in a nutshell when she states “…and after that came me” (pos. 45). This disassociation with their class social structure had important implications for the participants, who at times were treated as something abnormal or not human by their peers. This perhaps is most disturbingly exemplified by the stories of Carla, who experienced significant bullying by her peers which resulted in being physically endangered, who were likely influenced by the way in which her teacher viewed and treated her as unhuman. This finding, again, stems from the way her teacher treated her (as unhuman), and should perhaps be unsurprising, as prior work in this area of inquiry in different settings has demonstrated that the way in which teachers treat students with disabilities has a large influence on the way in which peers treat those same students [35].

In this respect, the problematic social hierarchies that were established within these PE classes were likely preventable, if teachers would provide accommodations to activities that encouraged or facilitated active and meaningful engagement for the participants. This recalls the second aspect of social hierarchy: the social hierarchy between students and teachers. With this in mind, we return to the narrative by Martha, who recalled receiving accommodations and the influence that the teacher could have on peers by explaining the need for these accommodations. This was, unfortunately, an exception among the participants, with whom many did not receive accommodations and their experiences were not enhanced by the behaviors of their teachers. Interesting, Martha also noted that her peers appeared to believe that she should not receive this special treatment, because it was an unfair special treatment for her advantage. In addition, several of the participants noted that they did not even view accommodations or changes in the curriculum as a possibility, and rather delegated the responsibility to create curricula that could be accessible to them to powers above the PE teacher. This leads to the third aspect of social hierarchy: the social hierarchy regarding power not directly lying in the hands of the social actors in PE lessons. This delegation of responsibility reinforces the idea that CWVI must simply attempt to ‘fit in’ to existing curricula, as there are no options for teachers to change curricula at all. Rather, and consistent with the extant literature [35], several participants’ experiences came to the same inevitable end: permanent removal from PE classes with their peers, and the eventual pursuit of education in other educational settings, which brought them to the setting of the current study.

In educational contexts, there is a clear hierarchy where teachers are positioned as authoritative figures who significantly shape what is valuable and desirable within the classroom [40]. For example, in PE classes where their teachers adopt competition-based cultures [41], it is not surprising that peers without disabilities may be reluctant to have CWVI on their teams because it would be a burden during competitive activities [42]. In cases such as this, students with disabilities are largely expected to ‘fit in’ to the curriculum and contribute to competition, which would generally be viewed through the lens of the supercrip phenomenon where the participants would overcome their impairments to excel in sport and inspire their classmates [43,44], or be exiled or discarded into isolated spaces and removed from activities because of their inabilities [43,45]. This appeared to be the case with the participants in this study, where teachers’ behaviors and a clear demonstration of a lack of value for the participants placed them into situations where they were unable to meaningfully participate in activities, which subsequently influenced their removal from activities, as well as their demotion in the social hierarchy among peers.

In the end, all of the participants reported an individual experience of a higher and ineluctable social hierarchy determining the specific social hierarchy within their PE class: the social hierarchy between peers is perceived as controlled by the teachers due to the authority given to them and the social hierarchy between the teachers and the students itself seems to be determined by external factors (curricula, school organization) lying out of reach for the actors in PE. As Carla states, this could even lie in the logic of society: “[…] in today’s society, where certain ideals simply prevail, I do not think it’s that easy to accept the other person as he or she is” (pos. 55). Eventually, the impression emerges that the CWVI interviewed had no independent way out from this situation. Entangled in such a complex system of social hierarchy, the participants searched for help in many places on different levels, since they talk with peers, ask teachers, go to the school’s principal and other representatives from school organization, talk with parents, and in Carla’s case even involve the police. However, in nearly all of these cases, the solution seemed not to be seen in the active search for a change of this problematic structure but rather in removing the “problematic” CWVI from the PE context or in not changing anything at all. Although one may question whether a single person can change such a complex system, fortunately enough, Martha’s story can be seen as a positive exception and gives a hint of how things can change within PE on a local level.

## 5. Conclusions

This study gives a deepened insight into four CWVIs’ perception of how PE settings they attended were socially constructed and their feelings of being included into these settings. First, the examination of the interviews shows that when being asked about feelings of being valued—which we used as indicator for feeling included—the respondents primarily reported negative feelings and experiences. In every interview we found moments of discrimination, exclusion or bulling, a perceived lack of understanding for the CWVIs’ situations, and little valuing of what they contribute to PE. Hence, a first result is that the interviewees did not seem to feel included in PE. The study further reveals that feelings of being valued were strongly linked to an underlying complex social hierarchy structure. The results made us aware of the fact that some kind of social hierarchy might emerge in every pedagogical setting. It became evident that it is not the setting per se that determined the social hierarchy, but that it is more about the concrete manifestation of social hierarchy and that this is obviously multi-layered and changeable. Within these contexts, the CWVI interviewed often relied on others for help. The expectation that teachers work to understand social hierarchies in their classes is therefore appropriate due to the existing authority relations in PE, and the finding that this expectation is often not fulfilled from the point of view of the interviewees is problematic. The study also reveals that—at least on the level of PE—change is possible. However, it must be remembered that teachers are also involved in social contexts and cannot simply ignore this or the curriculum. Existing ableist ideas, which can be found not least in German PE curricula [46], are thus presumably reinforced in many ways in this complex structure. Although these systems of hierarchy cannot simply be broken, against this background conscious, pedagogically responsible ways of dealing with one’s own ideas about body and performance [47] as well as an appreciative attitude towards all students on the part of the teachers seems to be of decisive importance.

Lastly, it should be noted that there are obviously limitations to this study. It should be mentioned that due to its design, we primarily interviewed students who left mainstream school and decided to attend a special school with boarding school accommodation. This preselection does not give a voice to students who saw no reason to leave mainstream school and who—perhaps—did not interpret their experiences in PE negatively, thus it also may indicate restricted results and show that additional research should be conducted. Consequently, further studies should survey more CWVI with additional disabilities in order to understand how experiences with comorbid conditions can influence experiences in PE.

## Figures and Tables

**Table 1:** Characteristics of the respondents (on the date of the interview).

Name	Age	Gender	Grade	Special School Since	Degree of VI	Additional Disabilities
Carla	20 years	Female	13th	11th grade	Blind	No
Jonas	19 years	Male	13th	11th grade	Visual impaired	No
Martha	19 years	Female	13th	11th grade	Visual impaired	No
Tim	19 years	Male	13th	5th grade	Blind	Hearing impairment

## Data Availability

The data presented in this study are available on request from the corresponding author.
